# Meeting the Needs of Emerging Adults With Type 1 Diabetes Living in a Rural Area With Mobile Health Interventions: Focus Group Study

**DOI:** 10.2196/55650

**Published:** 2024-08-07

**Authors:** April Idalski Carcone, Bree E Holtz, Madeleine Reardon, Dariane Vesey, Deborah A Ellis, Michael Parks

**Affiliations:** 1 Department of Family Medicine and Public Health Sciences School of Medicine Wayne State University Detroit, MI United States; 2 Department of Advertising + Public Relations College of Communication Arts and Sciences Michigan State University East Lansing, MI United States; 3 Nutrition and Wellness/Diabetes Education Upper Peninsula Health System – Marquette Marquette, MI United States

**Keywords:** emerging adults, type 1 diabetes, intervention, qualitative, mHealth, mobile phone, smartphone

## Abstract

**Background:**

Emerging adults (EAs; age 18-30 years) with type 1 diabetes (T1D) have more challenges with diabetes management and glycemic control than other age groups. Living in a rural community introduces additional unique diabetes care challenges due to limited access to specialty care and ancillary support services. Yet, few interventions have been developed to improve diabetes management in rural-dwelling EAs with T1D.

**Objective:**

This study aimed to understand the diabetes management experiences of older adolescents and EAs (age 16-25 years) with T1D living in a rural area and to assess their perceptions of the acceptability of 4 fully automated mobile health (mHealth) interventions to support diabetes management.

**Methods:**

EAs were identified by clinical staff through convenience sampling. In total, 8 EAs participated in 1 focus group and 1 EA completed an individual interview; all data were collected over Zoom. Facilitators explored EAs’ experiences living in a rural community with T1D and discussed EAs’ impressions of, feedback on, and recommendations for improving 4 mHealth interventions to meet the specific needs of EAs with T1D living in rural communities. Discussions were transcribed and analyzed using conventional content analysis.

**Results:**

In total, 9 EAs (aged 18.8, SD 2.7 years; 5, 56% men; 8, 89% White) with a duration of diabetes of 8.6 (SD 4.3) years participated. They described experiences with diabetes stigma (attributing diabetes to poor lifestyle choices) and feelings of self-consciousness (hyperawareness) in their rural communities. They attributed these experiences to the small size of their communities (“everyone knows”) and community members’ lack of knowledge about diabetes (unable to differentiate between type 1 and type 2 diabetes). In contrast, EAs reported high levels of social support for diabetes and diabetes care from family, friends, and other community members, but low support for medical needs. The location of their diabetes care providers and the limited accessibility of diabetes-specific and general medical care services in their local community created a challenging medical care context. Overall, EAs found mHealth interventions appealing due to their digital delivery and highlighted features that increased accessibility (voiceovers and simple, jargon-free language), individualization (ability to tailor intervention content and delivery), and applicability to their own lives and other EAs with T1D (relatability of vignettes and other content). EAs suggestions for improving the interventions included more opportunities to tailor the interventions to their preferences (greater frequency and duration, ability to adapt content to emerging needs), increasing opportunities for peer support within the interventions (friend and significant other as identified support person, connecting with peers beyond their local community), and making the tone of intervention components more casual and engaging.

**Conclusions:**

mHealth interventions aligned with EAs’ needs and preferences are a promising strategy to support EAs in communities where social support and resources might be limited.

**Trial Registration:**

N/A, not a clinical trial

## Introduction

Type 1 diabetes (T1D) impacts >1.6 million Americans, with >18,000 children and adolescents (<20 years) diagnosed annually [1]. Rural areas bear a higher burden of T1D (2.6 cases per 1000) compared to urban areas (2.2 cases per 1000) [2]. T1D care requires a complex daily regimen of blood glucose monitoring, tracking dietary intake, and administering insulin, with quarterly follow-up by diabetes specialty care providers [3]. However, <2% of endocrinologists practice in rural areas, with a third of people with T1D in rural areas without access to an endocrinologist within 50 miles of their home [4], and nearly two-thirds (62%) of rural counties lack diabetes support and education programming [5]. Youth with T1D in rural communities are less likely to have behavioral health providers among their diabetes care team [6] and are more likely to report poor patient-provider communication than their urban peers [7,8]. Thus, living in a rural community introduces barriers to an already challenging care regimen, which results in lower appointment keeping, more frequent hospitalization, and greater disease burden [9-13].

Emerging adulthood, the developmental period spanning the late teens to late 20s, is marked by increasing independence and autonomy, including transitions into new roles as emerging adults (EAs) enter college or the workforce [14,15]. This transitional period can further exacerbate the challenges of T1D management, as illustrated by EAs’ poorer diabetes management [16,17] and glycemic control [18] and higher rates of diabetic ketoacidosis [19]. EAs are less likely to use diabetes management technologies such as continuous blood glucose monitors (CGM) [20] and insulin infusion pumps [21]. The unique context created by the combined challenges of having T1D, living in a rural community, and being an EA underscores the need for interventions tailored to this high-risk population.

Studies investigating the specific needs of EAs with T1D in rural communities are limited, as is the availability of empirically supported interventions to support diabetes management in this population. Furthermore, existing behavioral interventions to support T1D management have mostly targeted children and younger adolescents; such approaches may lack developmental relevance for EAs [4,14,22]. Mobile health (mHealth) technologies are one strategy to deliver supportive interventions to improve diabetes management and health outcomes for EAs with T1D in rural areas [12-15]. In the US, nearly all adolescents have a cell phone by age 15 [23] and 96% of adults aged 18-29 have a smartphone [24]. Thus, cell phone delivered mHealth interventions are accessible to US EAs.

Our research team previously developed 4 mHealth interventions to support diabetes management among adolescents and EAs with T1D living in urban contexts. Each of these used a different evidence-based approach to improving illness management. These were (1) increasing the individual’s autonomy and self-efficacy for daily diabetes management [25], (2) providing reminders to complete diabetes care tasks [26], (3) improving communication with health care providers [27], and (4) enhancing support from family members to complete diabetes management tasks [28]. The motivation enhancement system (MES) is a brief counseling intervention to build intrinsic motivation and self-efficacy for daily diabetes management using Motivational Interviewing [29] techniques. TXT (SMS text message reminders) is a 30-day intervention to complete daily diabetes care tasks [30-32]. The question prompt list (QPL) facilitates patient-provider communication by helping EAs prepare a list of questions for diabetes medical appointments. MyT1DHero facilitates communication between adolescents and caregivers regarding daily diabetes management through education, gamification, and prompting. This study had two aims: (1) to understand the diabetes management experiences of EAs with T1D living in a rural area and (2) to assess EAs’ perceptions of the acceptability of these 4 different mHealth approaches to optimizing diabetes management.

## Methods

### Participants

Participants were recruited from the pediatric endocrinology department of a hospital located in Michigan’s Upper Peninsula. Michigan’s Upper Peninsula is composed of 15 counties, all of which are designated as rural by the US Census Bureau [33,34]. EAs meeting the following criteria were eligible: (1) age 16-25 years, (2) diagnosed with T1D for ≥6 months, (3) no comorbid medical conditions altering diabetes management, (4) no comorbid mental health conditions altering cognitive state, and (5) no learning disability precluding participation in study activities.

In total, 10 EAs were identified and agreed to participate; however, one did not complete the study. Of the 9 participants, most identified as non-Hispanic White (89%), half were male (56%), and the mean age was 18.8 (SD 2.7) years. In total, 6 (67%) were in high school, and 6 worked part-time. All 9 lived with their parents and rated their relative socioeconomic status as 5.6 (SD 1.4) out of 10 on the McArthur scale, reflecting a lower-than-average socioeconomic status [35]. The mean duration of diabetes was 8.6 years (SD 4.3) and most managed diabetes with a CGM (89%, n=8) and an insulin infusion pump (89%, n=8)*.* On average, EAs traveled 48.2 (SD 58.7) miles to attend a diabetes clinic at the recruitment site.

Study details are summarized in the CONSORT-EHEALTH (Consolidated Standards of Reporting Trials of Electronic and Mobile HEalth Applications and onLine TeleHealth) checklist available in Multimedia Appendix 1.

### Procedures

Participants were identified by a diabetes clinic nurse who reviewed their medical chart for inclusion. The nurse contacted eligible patients by phone to request permission to share their contact information with the research team. Research staff followed up with patients to explain study details and enroll them in the study. Eligible participants completed 2 study visits via Zoom. In the first, a research assistant obtained informed consent, after which participants completed a brief Qualtrics survey to record demographic information (eg, age, race, and gender) and diabetes treatment regimen. The second was the focus group discussion. One participant completed an individual interview.

### Focus Groups

In total, 2 focus groups with 3 and 5 participants each were led by 2 trained research assistants (RAs). One RA took notes and provided technological support. The second used a semistructured interview guide developed for the study to direct the discussions through 5 segments over 120-150 minutes. The first asked participants to describe their experiences living with T1D in a rural community, including barriers and challenges to diabetes management at the individual, family, peer, health care setting, and community levels. The next 4 segments introduced each intervention and solicited EAs’ impressions, feedback, and recommendations. One participant was unable to attend a focus group but agreed to complete an individual interview. This participant responded to the same questions from the focus group semistructured interview guide. The notetaker verified the Zoom focus group and interview transcriptions for accuracy.

### Interventions

#### Autonomy and Self-Efficacy Enhancement

The MES was 2 motivational interviewing-framed sessions. Session 1 began with psychoeducation about 3 key components of diabetes care, that is, glucose monitoring, insulin administration, and dietary management. Participants then advanced through motivation-enhancing exercises designed to increase intrinsic motivation and self-efficacy for diabetes management. It ended with setting a goal for daily diabetes care, that is, doing all diabetes care every day, doing more, and continuing to think about diabetes care (an autonomy-supportive option for those not ready to change their diabetes care behavior); behavioral strategies to support goal attainment, for example, setting reminders, establishing cues, and enlisting social support, were described. Session 2 reviewed progress toward the daily diabetes care goal, continued to grow motivation using reflection and motivation-enhancing exercises, and ended with a second goal-setting and behavioral strategy component.

#### Daily Reminders

*TXT* involved twice daily reminders to complete diabetes care tasks. Participants selected text reminders to complete 1 of 3 diabetes care domains (ie, insulin management, blood glucose monitoring, or dietary management) or selected a general reminder to “do their diabetes care.” TXT was further tailored to deliver reminders at times selected by the participant.

#### Communication With Health Care Providers

The *diabetes* *QPL* was developed to encourage active participation in diabetes clinic visits. The QPL was a list of diabetes care questions compiled through a literature search, web-based resources, and consultation with 3 diabetes medical care providers. QPL questions addressed common concerns in 3 diabetes care domains and EA-specific topics. The selected questions were emailed to the participant before their diabetes clinic visit.

#### Family Support

*MyT1DHero* aimed to improve communication and reduce conflict between adolescents (10-15 years old) with T1D and their caregivers. Designed using user-centered principles, the *MyT1DHero* app had 2 separate interfaces, 1 for adolescents and 1 for caregivers, which facilitated glucose testing via scheduling and tips on glycemic management. Adolescents and caregivers received 2-3 weekly prompts to encourage communication. Using the app earned adolescents points to “purchase” items for their superhero avatar, for example, capes, boots, logos, masks, and hair colors and styles. *MyT1DHero* also linked adolescents to peer video testimonials, sharing their stories and providing affirming messages. Information about *MyT1DHero* development and pilot study results can be found elsewhere [36-39].

### Analytic Plan

Data were hand-coded by 2 coding teams. The first was led by the lead author (AIC, female), a qualitative expert and developer of the MES, TXT, and QPL interventions, and included 4 coders, a paid RA, and 3 student volunteers. This team analyzed EAs’ feedback on living with T1D in rural communities and the MES, TXT, and QPL interventions using conventional content analysis [40]. During the initial training period, coders became familiar with the study aims and background, data collection methods, and analytic approach. They developed the initial coding framework based on the content of the interview guide and an initial review of all transcribed data. Coders independently coded each transcript, then met to review and discuss their coding, identifying and resolving coding discrepancies through discussion and collaboratively developing consensus-coded transcripts. The coding framework was refined and supplemented with examples throughout this iterative process. Data saturation was not a goal of this exploratory study. The secondary team coded feedback on *MyT1DHero* following a similar procedure. Coders were 2 RAs with previous experience with *MyT1DHero*, led by its principal investigator (BH).

### Ethical Considerations

The primary author’s institutional review board (IRB) approved all procedures (IRB protocol #071318B3E reviewed by the Wayne State University Institutional Review Board B3- Social, Behavioral, Education Committee [IRB00000327]). All research participants provided informed consent via an information sheet as part of the study enrollment process. For participants who were <18 years old, research staff obtained parental consent and youth assent. All study data were collected using a coded identifier to protect participants’ identities; the linkage to participants’ identities was stored securely and accessible only to IRB-approved research staff until the data analysis concluded, then it was destroyed. Participants received a US $50 gift card after the second visit.

## Results

### Diabetes Management in Rural Communities

EAs explained because they live in a small community “everyone already knows” they have diabetes, but people in their community do not understand the different types of diabetes: “It’s really hard to get anyone my age, or anyone in general, to understand the difference between type 1 and type 2. Even my really close friends still relate me to one of their grandparents that have type 2.” EAs reported that community members did not understand how they must live and care for their diabetes. This included voicing concerns about food and sometimes going even further and attempting to limit their eating behavior: “I deal with it every time there's a birthday or any sort of snack or something. People are like, can you eat that? Are you sure you can eat that?“ Because of this knowledge deficit, EAs reported experiencing *diabetes stigma*. “People just assume, ‘Oh, you didn’t take care of yourself, so you got diagnosed with diabetes.” These experiences contributed to *feelings of self-consciousness*. EAs reported feeling hyperaware or “on stage” when in a public context, leading to anxiety and sometimes changes in their diabetes care practices: “I wouldn’t leave class even though I could feel that my blood sugar was low because I didn’t want to have to ask.”

In contrast to those moments, EAs also recounted experiencing *diabetes-specific social support*. Not unexpectedly, family was a primary source of support for diabetes. EAs described their parents providing support in the day-to-day management of diabetes: “Sometimes, if I don't feel it [low/high blood glucose] or I don't notice, my dad's connected to mine, so he'll text me and let me know.” EAs reported that their parents also continued to play a key role in communicating with their diabetes care provider and coordinating their diabetes supplies: “My mom helps with the carb counting and servings, and, actually ordering the prescriptions and everything that I need.” Friends were another source of support, such as prompting EAs to check blood sugar after noticing a change in behavior or mood: “I have one best friend who if I'm mad or I'm upset or I'm going pale, she’ll be like, ‘what's your blood sugar right now?’” Finally, some described experiencing support from other members of the community. To illustrate, one EA explained, “I'm involved with a martial arts group that helps me take care of my diabetes a lot.”

Rural EAs described medical care challenges, including continuity of care, location and accessibility of diabetes care providers, and concerns about the affordability of medical care. For *continuity of care*, EAs’ discussed the challenge of trying to maintain the same team of medical care providers over time: “the hospital cycles through diabetes related personnel, every couple of years or sometimes even every year there’s new people.” EAs reported frequent changes in clinic personnel affected their relationship with providers: “For the past 5 years, I've had mostly the same diabetes doctor. But when I first got diagnosed, I saw all of them like one after the other and it felt like I was like resetting every single time because I would have to explain to them all these different things over and over again. By keeping with the same doctor, we can build upon things instead of restarting every time.”

Another challenge of clinical care in a rural setting was *provider location and accessibility*. EAs explained that they must travel long distances, which requires extended periods of time to receive their diabetes medical care: “For the past 14 years, I’ve had to drive 3 and a half hours for an endocrinologist, 4 times a year.” EAs reported limited access to diabetes support services in their local community: “It's a 2-hour drive there and at our clinic here there's not much diabetes education at all. We used to have a diabetic educator, but she retired and so there's no one.” EAs found accessing primary health care services was also a challenge: “I’ve been trying to see a primary care physician for the past year, just because everyone’s full up because there’s only so many doctors in the area. I’ve had to wait a while until the doctor will let me in.” Local providers were also unfamiliar with T1D: “a lot of people in the clinic, they know what it [T1D] is, but they don’t really know much about it.” EAs also described limited access to emergency services: “Back in 2020 something was wrong with my blood and I ended up passing out. I was seizing a little bit and we had to call the paramedics. Because we’re so far outside of town it probably took about 25 minutes for them to get to my house. I was fine but that was a realization for my family, that was pretty scary for us.” EAs also reported the scarcity of medical care impacted their transition to adult health care: “They very clearly said, we're not kicking you out, but you do have to look. You have to find someone else because their spots are limited too.”

Finally, EAs shared concerns about *medical insurance* and maintaining their current coverage: “I'm on Medicaid and every time I get my run down at the end of the quarter, I got that dread. There's no reason they should be kicking me off, but it's still that worry in the back of my head, giving me some anxiety.” They also voiced worries about insurance coverage after turning 26 and were no longer eligible for their parents’ insurance: “Having insurance is the biggest worry for all of us subconsciously. I originally went to school for pharmacy and now I switched over to education. Trying to focus on a career that has health insurance is huge. That’s everybody’s worry after 25.”

### Intervention-Specific Feedback

#### Building Autonomy and Self-Efficacy (MES)

Overall, EAs reported favorable impressions of the MES intervention. First, EAs found the intervention “user-friendly and straightforward” and viewed delivery between diabetes clinic visits as a strength. EAs appreciated features that enhanced the intervention’s *accessibility* to people’s different abilities. Specifically, EAs noted the avatar’s voiceovers eliminated barriers for people with different reading capabilities: “This is a problem I used to have when I used to play video games and I couldn’t read, or when I could barely read. I didn’t know what was happening.” They also noted the use of simple language versus technical jargon made the interventions appropriate for broad audiences, with several specifically noting those newly diagnosed with T1D: “There wasn’t a lot of jargon or anything in the video, so it just seemed simple and straightforward. Something that a new diabetic in particular would be comfortable using.” They appreciated the collaborative, nonjudgmental nature of the intervention: “it seems to not talk down on you, it’s more like we can work to better your management.”

EAs found the MES content *applicable* to their own lives and others with T1D: “A lot of diabetics can relate to having their high hemoglobin A_1c_ and growing from that and looking back and realizing how many changes you’ve made and how they’ve really helped you.” EAs found the vignettes illustrating behavioral strategies to support the completion of daily diabetes care helpful, especially those leveraging features of diabetes technology. They recommended including more tips, advice, and education to maximize technology use to support diabetes care, such as calendar alerts programmed into a smart phone and websites to facilitate carbohydrate counting: “They do sound really, really helpful. I know that I should start implementing some strategies to remember more things.” EAs had several *suggestions for improvement*, including finding ways to make the intervention more engaging, including more conversational peer testimonials [“more like heart-to-heart” or “interview style”], more charismatic actors, and making the tone of the intervention more casual and engaging: “It just felt really professional. You were getting a talking to.” Finally, EAs suggested adding components to address the “mental part” of living with diabetes, including coping with feelings associated with stigmatization and self-consciousness, and exercise, “that can be really scary, if you’re worried about your blood sugar jumping or going down super low.”

#### Reminders for Diabetes Management (TXT)

Overall, EAs found the TXT intervention acceptable, indicating the intervention was a good fit for their age and use of technology: “I’d definitely use it.” EAs described the reminders as a “gentle nudge” and the integration of the participant’s name into the message as “more personal.” EAs appreciated that TXT content was *individualized* to their preferences and diabetes treatment regimen. EAs liked being able to tailor the timing of the reminders to times they often forget to complete their care or higher-risk times: “I like that you can choose what time because I personally have certain times where I know I’m usually going to be at a higher blood sugar.” They also appreciated being able to tailor the content of the messages and suggested more tailoring options: “If you had a checklist of what [diabetes technology] you’re using.” EAs liked the variation in the wording of the messages: “I really like that it isn’t the same message over and over. There’s some difference in some of the messages to you’re not just getting the same sentence over and over, and then you start to ignore it.” While most thought the reminders would be useful, some did note they might be redundant or unnecessary depending on the diabetes technology an individual might be using: “With my [continuous glucose monitor], I’m reminded to check my blood sugar as often as I need and if my blood sugar is high, my pump will vibrate.” EAs thought TXT might be particularly useful for those newly diagnosed or undergoing a life transition: “the reminders would be more useful for someone who is getting back into a routine, like going to college.”

EAs found the intensity of the reminders acceptable but suggested greater intensity might be welcome. Specifically, 2 texts per day was “a pretty good number, one in the morning, one in the night, as a general reminder to do important things,” but EAs suggested 4 [“breakfast, lunch, dinner, and then make sure your blood sugar is on track before you go to bed”] or 5 [“morning, because you’ve had breakfast, and then lunch, and I have a snack between lunch and dinner, that would be another good time, then dinner and then late at night”] would fit better with their needs. EAs cautioned, “more than five would be a lot” and “overdoing it.” EAs thought “30 days is a pretty good amount of time to at least start a habit and get into a routine” but suggested offering the option to extend the intervention “until you had enough.” EAs also suggested greater flexibility in tailoring the content and timing of the reminders over time and to restart the reminders later: “People should have the option whether they want to continue what they are doing or if they need help at a different time. Being able to switch when you get the reminders would be really helpful.”

#### Question Prompt List (QPL)

Overall, the QPL intervention was well-received and acceptable. EAs described it as helpful “because sometimes I forget what questions I was going to ask” and a useful prompt for topics “you might not have even considered before.” EAs thought the QPL might be particularly helpful during times of transition: “I probably would have appreciated [it] a few months ago when I was in my process of getting my [continuous glucose monitor] because that was a lot of questions in a short amount of time.” Finally, EAs thought the scope of the content sufficiently broad: “questions were good for beginners and there were also questions that were a little more in detail, things that I would ask at appointments.” EAs appreciated the *individualization* of the question list to their diabetes treatment regimen and the ability to write their own questions: “where you could personalize your own questions and have them send it to you.” They also appreciated the component of the intervention dealing with age-appropriate concerns such as driving: “Once you hit a certain age, you check [blood glucose] before you drive and stuff like that. So, having those questions on there would be nice for kids who maybe might not feel comfortable talking to their parents about it.” There was one content suggestion, to include a prompt about prescription renewals.

When it came to delivering the QPL, EAs thought having their list of questions sent to them digitally (via email, text, or an app) was best: “then you can just pull it up” but noted having the ability to “take notes in the margins would also be helpful.” Some thought integration with their patient portal would help facilitate communication between quarterly clinic visits, although some topics raised concerns about confidentiality, “You have to make sure that all that information is between who you want to be between” or justified a more in-depth, face-to-face conversation: “Questions about correction factors could probably be over the portal, but if I’m like, ‘how do I do marijuana while taking care of my blood sugar, that’s probably a full conversation with the doctor.” EAs also thought it would be useful to have a copy sent to their diabetes care team.

#### MyT1DHero

Overall, EAs responded positively to MyT1DHero and thought it would promote communication and independence in diabetes management: “It’s hard to transfer over from having a parent controlling everything to having more control over it yourself. Having that communication and shared information without needing to constantly be texting or calling or anything would be incredibly handy.” EAs suggested that the app could be improved in 3 ways. First, EAs suggested enhancing social support features to allow a friend, roommate, or significant other to be the support person rather than a parent: “You'd want to add someone else, someone you're living with.” They also highlighted the potential for the app to provide a broader base of social support. Noting feelings of isolation and stigmatization due to a lack of peers with T1D in their community, EAs believed incorporating a social support component that connected them with peers outside of their rural community would provide a critical source of support: “Knowing there are other people who are having the same problems. It's not like the world has singled you out for some reason.”

## Discussion

### Principal Results

This study examined the experiences of a high-risk population of EAs with T1D living in rural communities [14,15]. EAs reported diabetes challenges in their community and medical care. Firstly, EAs felt stigmatized because of their diabetes. More than half of adolescents [41] and EAs [42] with T1D report feeling stigmatized in their communities. Adults with diabetes living in rural communities also report greater stigma than those in non-rural settings [43]. The visibility of diabetes wearable technologies, that is, pumps and CGMs, may contribute to the experience of stigma [43]. Diabetes stigma is associated with poor diabetes quality of life [41], poor glycemic control [42], health complications [44], and poor psychosocial functioning [45], which were also reported by rural EAs in this study. While mental health screening and referral to behavioral services are standard practices [46,47], the assessment of stigma and its negative sequelae may be underrecognized. Conversely, rural EAs also reported perceiving social support related to caring for their diabetes from family, friends, and other trusted community members, a protective factor that may offset the detrimental effects of stigma [48]. Positive role models who provide health advice can be a critical source of social support for youth [49], but may be limited in rural communities [49].

EAs described challenges in their diabetes medical care due to traveling long distances to receive medical care and a limited number of providers in their local community, which impacted the continuity and quality of their diabetes medical care. These concerns are consistent with previous reports of reduced access to health care providers and specialty care providers specifically [4,10,11,49], frequent provider turnover [10], and limited resources for diabetes education [12] and ancillary services [7,8]. While telehealth may be a useful supplement to in-person care, EAs in other contexts have stressed the importance of in-person care for high-quality diabetes care [50]. EAs are also worried about their pending transition from the pediatric to adult health care system and their ability to afford medical care. A T1D Exchange study [51] found financial stress to be a significant predictor of having an elevated hemoglobin A_1c_ amongst EAs with T1D, suggesting a need for supportive interventions during this transition.

Despite the elevated risk experienced by EAs with T1D living in rural communities with scarce resources for diabetes management, few interventions have been designed specifically to meet the needs of this population. EAs themselves have reported that the transition to adulthood and the assumption of responsibility for diabetes care are significant challenges [52], reinforcing the need for support during this developmental period. The EAs in this study viewed the mHealth interventions examined as user-friendly and acceptable. They identified strengths and weaknesses of the reviewed interventions that could be applied to future interventions developed for the EA population.

As digital natives, EAs are early adopters, savvy users, and innovators of technology use, having grown up with technology integrated into their social ecology [53]. Thus, it is unsurprising that EAs described technology-delivered interventions as a good fit for their age group and recommended further integration with technology, such as linking intervention components to the electronic medical record. For EAs living in rural communities, technology-based interventions may offer critical support in a setting where resources are low and barriers to supportive services are great [54].

In addition to digital delivery, EAs highlighted aspects of the interventions’ that increased accessibility, individualization, and applicability of the intervention content to their own lives and those of other EAs living with T1D. A recent systematic overview of 18 systematic reviews and meta-analyses of digital interventions for adolescents and young adults with mental health conditions similarly found design elements that were credible, engaging and interactive, relatable, and flexible were associated with acceptability [54]. In addition to being acceptable, mHealth interventions demonstrate promise for improving glycemic control [55,56].

Consistent with their developmental stage, EAs found aspects of the intervention that supported their independence appealing and suggested ways to further support their growing independence. For example, they suggested augmenting the MyT1DHero intervention’s communication feature to allow for friends, roommates, or significant others to support their diabetes care rather than exclusively relying on parental support. Additionally, EAs recommended that the intervention broaden its social support component by facilitating connections with peers beyond their local community. They believed that expanding their social network in this manner would help mitigate the feelings of isolation and stigma often associated with T1D. Other studies conducted with urban adolescents have supported the value of peer support beyond one’s local community [57,58].

### Limitations

This study has limitations. First, EAs in this study were all using state-of-the-art diabetes care technology (ie, insulin pumps and CGMs) and self-described themselves as well-managed and independent in their diabetes care, which differs from reports that EAs are less likely than other age groups to use diabetes technologies consistently [20,21] and more likely to experience suboptimal adherence [16,17], glycemic control [18], and higher rates of diabetic ketoacidosis [19]. Furthermore, they were all from a small rural area of Michigan. Thus, their diabetes care experiences, needs, and perceptions of the interventions presented may differ from those of EAs not using the same diabetes technology, with the same high level of glycemic control, or living in different rural communities. A methodological limitation may be the use of video conferencing to conduct the focus groups. While video conferencing enabled EAs living in geographically distant communities to come together for the focus groups, the dynamics of the discussions may have been attenuated in the digital domain, although others have found no impact on data quality [59]. Finally, given the limited sample and small number of focus groups, these results may not reflect data saturation.

### Conclusions

mHealth intervention is a promising strategy to support EAs with T1D living in rural communities. EAs found a variety of approaches to optimizing diabetes management acceptable and well-aligned with their developmental needs for autonomy and independence. Each approach was seen as offering critical support for diabetes management in communities where social support and tangible resources were limited. Their feedback highlights the importance of tailoring supportive interventions to the specific needs and preferences of EAs in rural communities [60,61]. Next steps could include refining these intervention approaches based on this feedback and testing their impact on diabetes management and glycemic outcomes among EAs living in rural contexts.

## Appendix

Multimedia Appendix 1 This file contains the authors' responses to the CONSORT-EHEALTH (Consolidated Standards of Reporting Trials of Electronic and Mobile HEalth Applications and onLine TeleHealth) checklist.

## Abbreviations

CGM: continuous blood glucose monitors
CONSORT-EHEALTH: Consolidated Standards of Reporting Trials of Electronic and Mobile HEalth Applications and onLine TeleHealth
EA: emerging adult
IRB: institutional review board
MES: motivation enhancement system
mHealth: mobile health
QPL: question prompt list
RA: research assistant
T1D: type 1 diabetes
TXT: SMS text message reminders

## Data Availability

The data sets generated analyzed during this study are available from the corresponding author after the execution of a data use agreement.
